# Reducing culture underestimation in urine: flow cytometry–guided validation of low-dose DTT pre-treatment for biofilm dispersal

**DOI:** 10.1128/spectrum.03910-25

**Published:** 2026-05-05

**Authors:** Luigi Regenburgh De La Motte, Ivan Muradore, Fabiana Giarritiello, Erica Stefàno, Loredana Deflorio, Lorenzo Drago

**Affiliations:** 1 UOC Laboratory of Clinical Medicine with Specialized Areas, IRCCS MultiMedica, Milan, Italy; 2 F.L.O.R.A.L. facility IRCCS MultiMedica, Milan, Italy; 3 Department of Medicine and Health Sciences “V. Tiberio”, University of Molise 18960, Campobasso, Italy; 4 Department of Biomedical Health Sciences, University of Milan 9304, Milan, Italy; Reichman University, Herzeliya, Israel

**Keywords:** urinary tract infections, flow cytometry, biofilm, dithiothreitol, diabetes

## Abstract

A substantial proportion of symptomatic patients with urinary tract infections (UTIs) have negative or low-count urine cultures despite clinically compatible presentations. Biofilm-associated bacterial aggregation and the presence of viable bacteria not recovered by standard culture may contribute to this diagnostic gap. Flow cytometry (FACS) can detect these viable cells, but culturability remains essential for species identification and antimicrobial susceptibility testing. We evaluated whether low-concentration dithiothreitol (DTT) can safely disperse urinary biofilm-associated aggregates and improve bacterial recovery from urine samples. Seventy-two clinical urine samples were processed in parallel (untreated vs 0.1% DTT-treated). Viability was quantified using SYTO9/PI-based flow cytometry with bead-normalized bacterial counts. Culturability was assessed on chromogenic media. Comparative analyses included paired statistics, Bland–Altman agreement, and correlation between viable and culturable fractions. DTT (0.1%) did not alter bacterial viability (*P* = 0.47), confirming its non-toxicity at this concentration. In contrast, DTT significantly increased CFU recovery (median: 10 to 5.5 × 10³ CFU/mL; *P* < 0.0001), converting several previously negative or low-count samples into positive cultures. Agreement between viable and culturable fractions improved after DTT treatment, primarily through reduced systematic bias in Bland–Altman analysis, while correlation coefficients remained similar (r: 0.44 vs 0.45). A persistent discrepancy between FACS-derived viable counts and culture-based counts suggests the presence of viable bacterial populations not fully recovered by standard culture conditions, findings that are consistent with but do not directly demonstrate the presence of VBNC subpopulations. Low-dose DTT is a safe and effective pre-analytical treatment that markedly improves culture sensitivity without compromising bacterial viability. These findings support further investigation of DTT-assisted processing as a potential strategy to reduce culture underestimation in urinary diagnostics and to better characterize viable bacterial populations not detected by standard culture methods.

**IMPORTANCE:**

Standard urine culture may fail to detect the full spectrum of bacteria present in urinary tract infections, particularly when microorganisms are embedded within mucus, biofilm-like aggregates, or enter a viable but non-culturable state. These hidden bacterial populations may contribute to persistent symptoms or recurrent infections while remaining undetected by routine culture methods. Our findings suggest that pre-treating urine samples with a low concentration of dithiothreitol may help disperse biofilm-like aggregates while preserving bacterial viability. When combined with flow cytometry, this approach may allow a more comprehensive estimation of the viable bacterial fraction and, in some cases, may increase the likelihood of bacterial recovery by culture in samples that would otherwise appear negative. Because the procedure relies on inexpensive reagents and techniques compatible with routine laboratory workflows, it may represent a practical adjunct to conventional diagnostics. Further studies are needed to confirm its clinical impact.

## INTRODUCTION

A foundational challenge in microbiology—from clinical diagnostics to environmental monitoring—is the inability of standard culture techniques to accurately quantify the true viable bacterial population. Many organisms persist in aggregated forms, such as mucus-embedded clusters or structured biofilms (1–3), or may enter a viable but non-culturable (VBNC) state (4–6), both of which can impair growth on artificial media. The result is a well-recognized diagnostic discordance in which viable bacteria may remain undetected by routine culture, limiting sensitivity and contributing to missed or delayed diagnoses (7).

This challenge is particularly evident in urinary tract infections (UTIs), where 15%–25% of symptomatic patients present with negative or low-count cultures despite compatible clinical features (8, 9). These so-called “culture-negative” UTIs are unlikely to be truly sterile; molecular and cytometric studies demonstrate that viable bacteria can persist within mucinous aggregates or early biofilm-like structures that may limit release during routine sample processing (10, 11). These limitations of standard culture methods can contribute to diagnostic uncertainty in clinical practice (12).

Several technologies have attempted to address this gap. Molecular assays detect microbial DNA with high sensitivity but cannot discriminate between viable and non-viable organisms (8, 13). Flow cytometry (FACS), conversely, quantifies viable cells at the single-cell level but cannot provide species identification or antimicrobial susceptibility testing, which remain culture-dependent (14). Thus, a critical unmet need persists for a simple, scalable pre-analytical strategy that can physically disperse aggregates, restore culturability, and maintain cell viability.

Dithiothreitol (DTT) is a mucolytic and disulfide-reducing agent widely used to liquefy respiratory secretions and disrupt biofilms. In urine, mucin represents a major structural component capable of entrapping bacteria and reducing plating efficiency (15). We hypothesized that low-concentration DTT could act as a dispersing agent, releasing viable bacteria from mucus and biofilm matrices and thereby bridging the diagnostic gap between FACS-detected viability and culture-based identification.

Here, we validate this approach using a parallel analytical design in 72 clinical urine samples. By comparing FACS-derived viable counts with culture-based colony recovery before and after DTT exposure, we evaluate whether low-dose DTT enhances microbial dispersion and improves bacterial recovery without compromising viability. These findings support the potential value of this simple pre-analytical strategy for improving bacterial recovery from urine samples and for better characterizing discrepancies between cytometric viability and culture-based detection (16, 17).

## MATERIALS AND METHODS

### Sample collection

Urine samples were collected from hospitalized and ambulatory patients at the IRCCS MultiMedica network, which includes the Sesto San Giovanni, San Giuseppe, and Castellanza hospitals (Milan, Italy), covering a broad geographic area surrounding Milan. Samples were processed following the routine diagnostic workflow for urine culture according to Italian AMCLI (Associazione Microbiologi Clinici Italiani) guidelines. A residual portion of each specimen (post-diagnostic discard) was anonymized and used for experimental analysis.

### DTT preparation and urine pre-treatment protocol

Dithiothreitol (DTT) was obtained from Biolife Italiana S.r.l. (Milan, Italy) as Sputafluïd Pastilles. A 0.1% (w/v) solution was freshly prepared by dissolving 0.1 g of powder in 100 mL of sterile distilled water immediately before use. For each experiment, 10 mL of urine was centrifuged at 3,000 × *g* for 3 min; the supernatant was discarded, and the pellet was resuspended in 1 mL of 0.1% DTT solution. Samples were incubated for 15 min at room temperature under gentle agitation to promote the dispersion of mucus and biofilm matrices while preserving bacterial viability. After incubation, each suspension was divided into two aliquots: one for flow cytometric analysis and one for plate culture. Untreated urine aliquots served as controls.

### Flow cytometry

Bacterial enumeration and viability were assessed by analytical flow cytometry (FACS) using BD LSRFortessa X-20 flow cytometer (BD Biosciences, San José, CA). Samples were stained with the LIVE/DEAD BacLight Bacterial Viability and Counting Kit (L34856 Thermo Fisher Scientific, USA), following the manufacturer’s instructions. Briefly, 3 µL of fluorescent dye mixture (SYTO9 and propidium iodide) and 10 µL of microbeads were added to 1 mL of bacterial suspension and incubated in the dark for 15 min at room temperature. Data were collected using forward scatter (FSC) and side scatter (SSC) parameters for bacterial gating, and green (SYTO9) and red (PI) fluorescence channels to distinguish live (SYTO9^+^/PI⁻) from dead (SYTO9^+^/PI^+^) cells. A minimum of 10,000 events was acquired per sample, and viable cell counts (cells/mL) were calculated by normalizing the number of total beads counted in the samples. Controls (unstained, heat-killed, and single-stained samples) were used for spectral overlap compensation and gating calibration. Flow cytometry data were collected and exported to FCS Express 7 (*De Novo* Software, Los Angeles, CA) for post-acquisition processing.

### Plate culture and colony enumeration

In parallel, 1 µL of each DTT-treated and untreated suspension was inoculated onto chromogenic agar plates (ChromID CPS Elite, bioMérieux, France) and incubated for 12 h at 37°C under aerobic conditions, corresponding to the early diagnostic readout routinely used in our clinical laboratory workflow, following AMCLI guidelines. Chromogenic agar (ChromID CPS Elite) allows presumptive differentiation of common uropathogens based on colony color; however, species-level identification was not systematically performed for all isolates within the scope of this analytical study, as the primary objective was to quantify differences in bacterial recovery between untreated and DTT-treated samples. Colony-forming units (CFU/mL) were determined by direct counting. Plates yielding <10 CFU/mL were interpreted as negative, according to clinical laboratory criteria. Comparative analyses were performed between No DTT and +DTT aliquots to assess the effect of DTT treatment on bacterial recovery and culturability.

### Statistical analysis

All quantitative data were expressed as median and interquartile range (IQR) unless otherwise specified. Bacterial counts were log₁₀-transformed prior to analysis to normalize distributions and enable cross-method comparison. Differences between paired aliquots (No DTT vs +DTT) were analyzed using the Wilcoxon signed-rank test. Correlations between FACS and plate culture data were evaluated using both Pearson’s correlation coefficient (r) and Spearman’s rank correlation (ρ). Agreement between analytical and culture-based quantification was assessed using Bland–Altman plots, estimating mean bias and limits of agreement. For culture-negative samples (CFU = 0), a constant value of 1 CFU was added prior to log10 transformation to allow statistical comparison. For cross-method analyses (FACS vs CFU), samples with FACS counts below the quantification limit were excluded (Live = 1 a.u.). This resulted in *N* = 69 for No-DTT (three samples) and *N* = 71 for +DTT (one sample). All 72 pairs were retained for within-method comparisons (No-DTT vs +DTT) where applicable. All analyses and plots were performed using GraphPad Prism v8.0 (GraphPad Software, USA). A two-tailed *P*-value < 0.05 was considered statistically significant.

Raw paired FACS and culture data used for all statistical analyses are provided in Data set S1

## RESULTS

### Effect of DTT on bacterial viability assessed by flow cytometry

**Fig 1:**
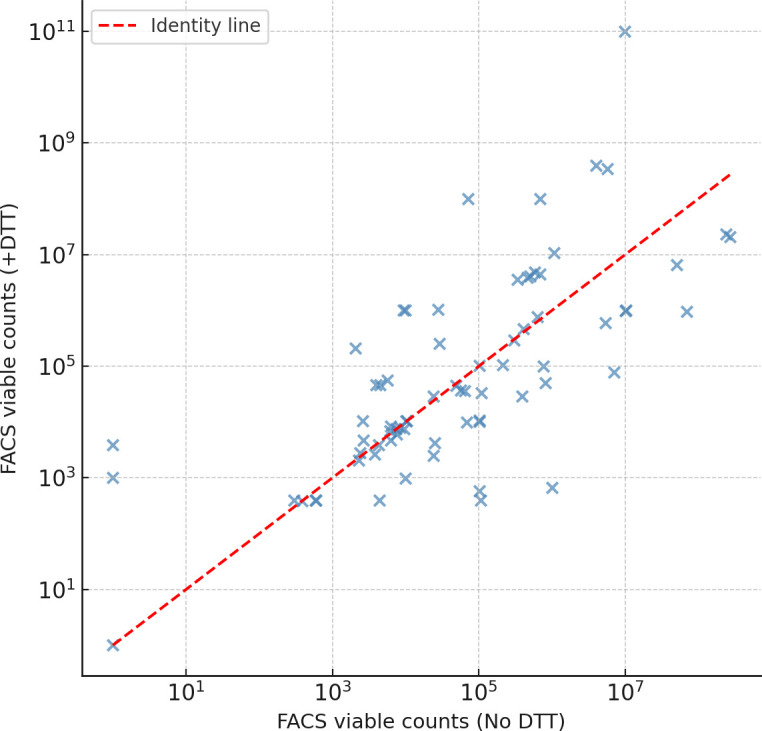
Effect of DTT on bacterial viability assessed by flow cytometry. Scatter plot of viable bacterial counts (log₁₀ cells/mL) obtained by FACS before and after 0.1% DTT treatment. The dashed line represents the identity line (y = x). No significant difference was observed, indicating that DTT does not alter bacterial viability.

A total of 72 paired urine aliquots were analyzed by analytical flow cytometry (FACS) before and after DTT treatment. The distribution of live bacterial events remained comparable between the two conditions, with median counts of 7.45 × 10³ (No DTT) and 7.72 × 10³ (+DTT). Statistical testing confirmed the absence of significant differences (parametric *P* = 0.546; non-parametric *P* = 0.470), indicating that exposure to 0.1% DTT—a concentration known to effectively disrupt biofilm matrices—did not induce measurable cytotoxicity in urinary bacterial populations. The Bland–Altman analysis revealed a minimal bias close to zero, suggesting that observed variability reflected sample heterogeneity rather than treatment-related cell loss. These data indicate that DTT is chemically compatible with bacterial viability during pre-analytical urine processing, preserving bacterial integrity and viability during cytometric analysis. This aspect is crucial when adapting flow cytometry for clinical diagnostics, ensuring that biofilm dispersal does not compromise bacterial detection accuracy (Fig. 1).

### Plate culture response to DTT treatment

**Fig 2:**
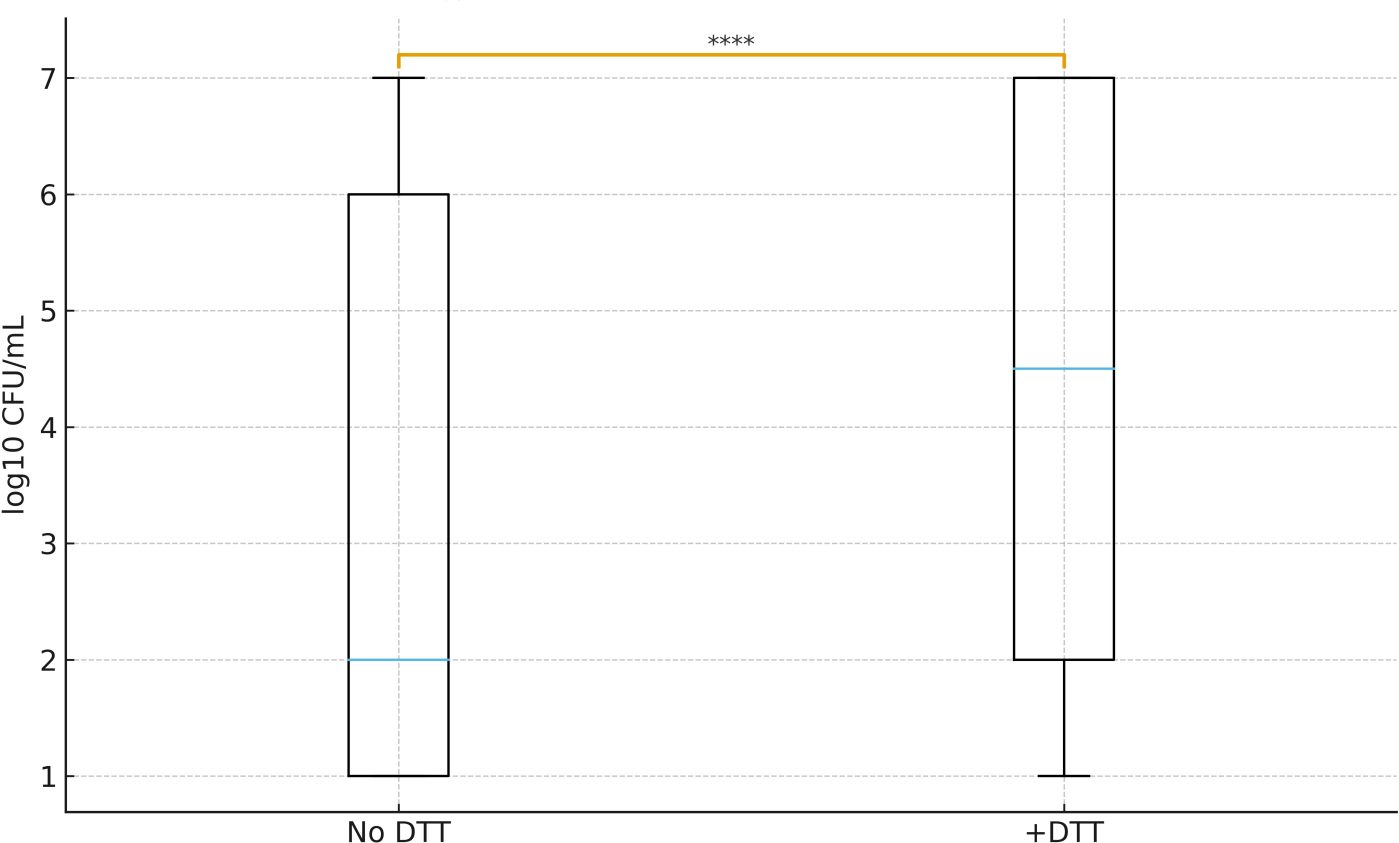
DTT enhances bacterial recovery in plate culture. Boxplots of CFU/mL before (No DTT) and after DTT treatment. DTT significantly increased colony recovery (Wilcoxon *P* = 1.6 × 10⁻⁵). Boxes represent the IQR, horizontal lines the medians, whiskers 1.5× IQR. ****P < 0.0001.

When the same urine aliquots were cultured, a marked increase in bacterial recovery was observed following DTT treatment. Median colony-forming unit (CFU) counts rose from 10 CFU/mL (No DTT) to 5.5 × 10³ CFU/mL (+DTT), with a Wilcoxon paired test *P*-value of 1.6 × 10⁻⁵. Several previously negative or low-count samples became culture-positive after DTT exposure, suggesting that DTT released bacteria trapped within mucus aggregates or early biofilm structures. This increase in culturability may have diagnostic implications, as low-yield or negative cultures can complicate microbiological interpretation in routine practice. By enhancing microbial dispersion and plating efficiency, DTT may improve bacterial recovery from samples where microbial aggregates limit culture detection, especially in patients with biofilm-associated, chronic, or partially treated infections (Fig. 2).

### Cross-method comparison between flow cytometry and plate culture (No DTT)

**Fig 3:**
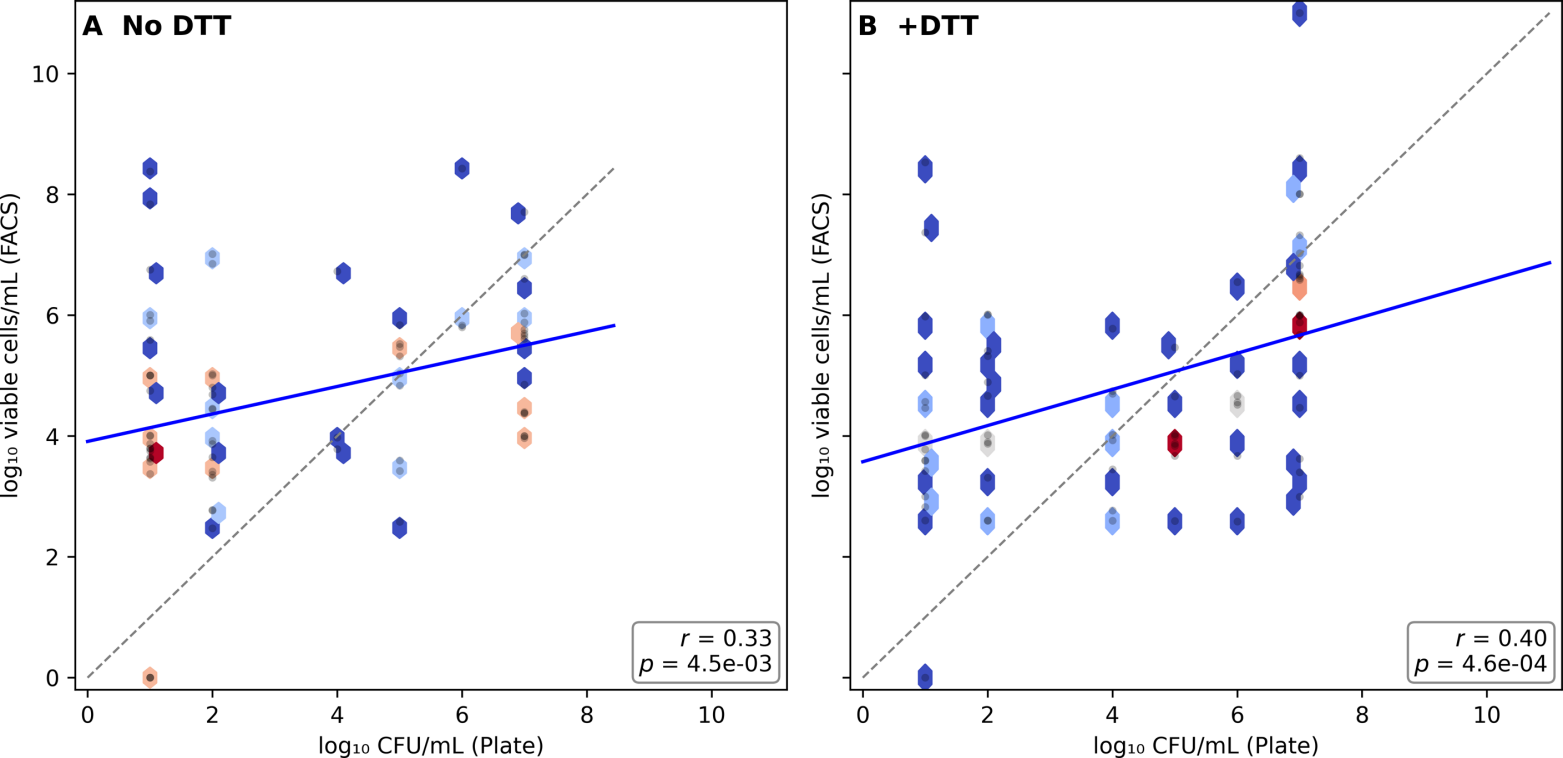
Correlation between flow cytometry and plate culture counts. (**A**) Untreated samples and (**B**) DTT-treated samples plotted as log₁₀ FACS vs log₁₀ CFU/mL. Dashed line: identity line (y = x). Solid line: linear regression. Agreement improved mainly through reduced Bland–Altman bias, while correlation coefficients remained similar.

In untreated urine samples (*N* = 69), flow cytometry consistently yielded higher bacterial counts than plate culture, with a median log₁₀ difference of +1.81 (≈65-fold higher) in favor of FACS (*P* = 2.0 × 10⁻⁶). Correlations between the two methods were moderate but significant (Pearson r = 0.45; Spearman ρ = 0.48; both *P* < 0.001), confirming a consistent relationship but distinct analytical sensitivity. Bland–Altman analysis showed a positive bias of +1.22 log₁₀, indicating that flow cytometry systematically detected higher bacterial counts than plate culture. The limits of agreement ranged from −3.60 to +6.03 log₁₀, reflecting substantial variability between the two methods. After DTT treatment, the mean bias decreased to +0.64 log₁₀, indicating improved agreement between FACS and culture. The limits of agreement ranged from −3.96 to +5.24 log₁₀. This reduction in systematic bias, rather than changes in correlation coefficients, indicates that DTT improves agreement between viable and culturable fractions primarily by increasing colony recovery. These results are consistent with the presence of bacterial populations that remain viable but are not recovered by standard culture conditions, a phenomenon often associated with viable but non-culturable (VBNC) states that maintain metabolic activity but fail to replicate on conventional media. Such cells are common in urine following antibiotic exposure, osmotic stress, or biofilm adaptation, and their underestimation by culture may contribute to discrepancies between cytometric viability and culture-based detection (Fig. 3).

### Cross-method comparison in DTT-treated samples

**Fig 4:**
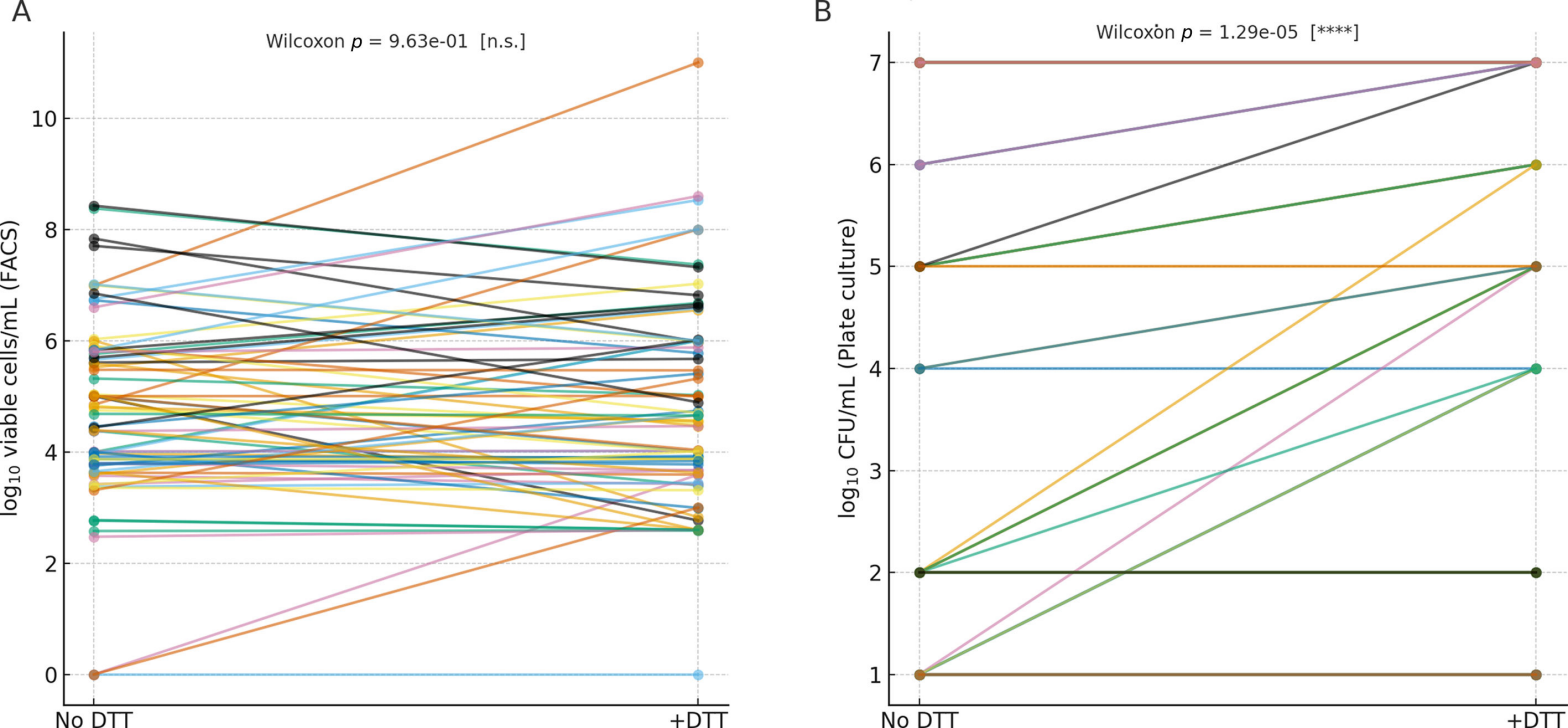
(**A**) Paired FACS counts before and after DTT. Paired log₁₀ viable counts obtained by FACS for each sample. No significant difference was observed, confirming that DTT does not affect viability. (**B**) Paired plate culture counts before and after DTT. Paired CFU/mL values obtained by culture. A significant increase was observed after DTT addition, confirming its effect on culturability. ****P < 0.0001; n.s., not significant.

After DTT exposure (*N* = 71), the difference between FACS and plate culture decreased but persisted. FACS counts remained on average 0.5 log₁₀ higher than plate CFU values (*P* = 0.0053), reflecting a partial convergence between analytical and culture-based methods. Correlations between the two techniques remained significant (Pearson r = 0.44; Spearman ρ = 0.47; both *P* < 0.001), supporting improved agreement following biofilm dispersal. These results suggest that DTT enhances bacterial culturability and reduces the analytical gap between cytometric and culture-based quantification. However, a measurable fraction of cells remained detectable by FACS alone, consistent with residual viable populations not recovered by culture, potentially including VBNC-like states that, while viable, cannot resume replication. Thus, FACS provides complementary diagnostic value by revealing the total viable bacterial burden, whereas culture identifies the clinically actionable culturable fraction (Fig. 4).

### Clinical interpretation and summary of findings

**Fig 5:**
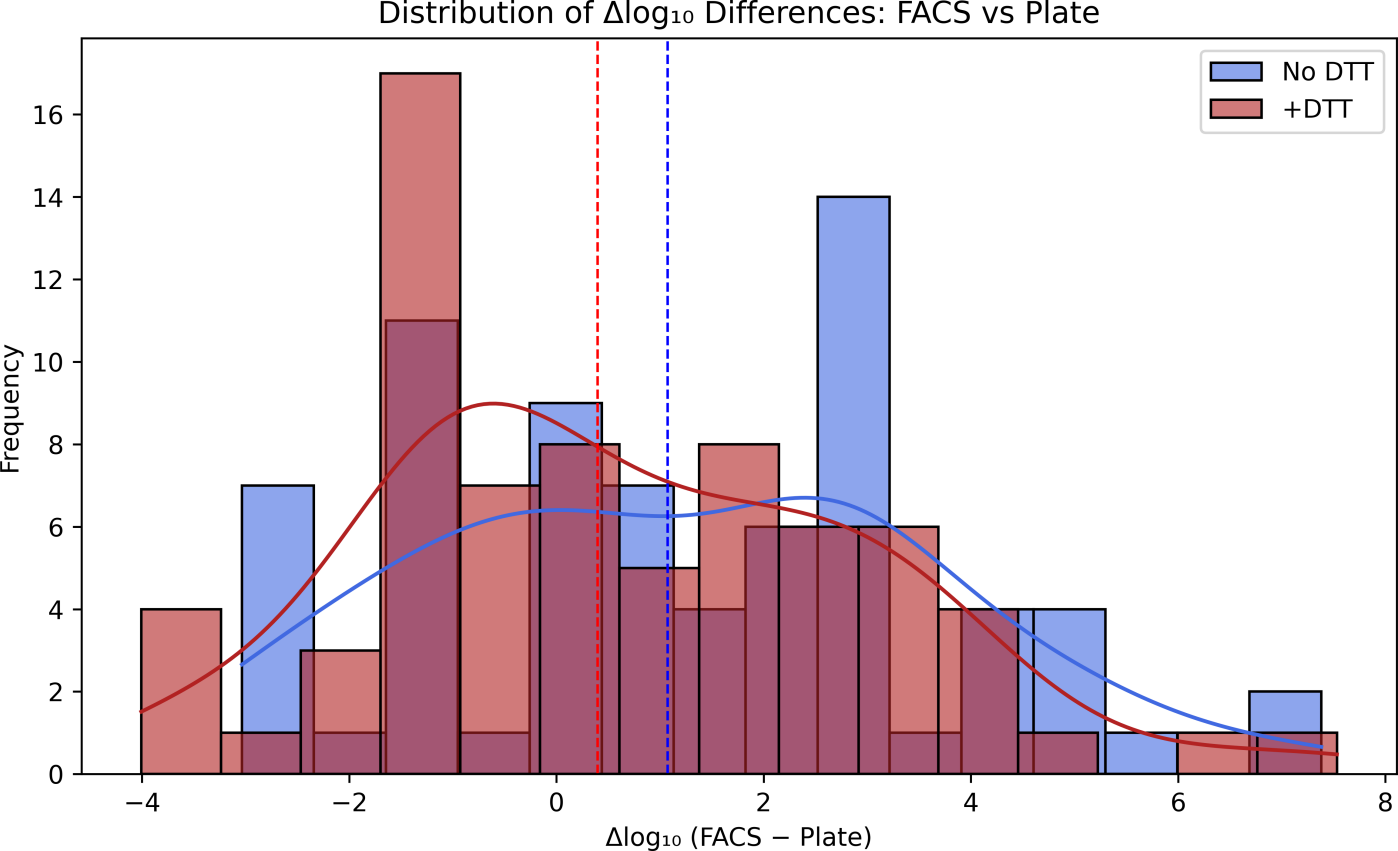
Distribution of Δlog₁₀ (FACS – Plate) differences. Histograms comparing Δlog₁₀ values before and after DTT. Positive values indicate viable but non-culturable fractions. DTT narrows the distribution, indicating improved agreement between FACS and culture.

The overall findings presented in Table 1 reveal a clear and consistent trend across all analytical approaches. Flow cytometry systematically detects a higher number of viable cells compared with conventional culture, indicating that a proportion of living bacteria remains unculturable under standard laboratory conditions. When DTT is introduced, this discrepancy begins to shift: culture recovery increases markedly, while FACS viability estimates remain essentially unchanged. This pattern strongly suggests that DTT facilitates the release of bacteria that were physically trapped within urine-associated matrices or early biofilm structures, restoring their ability to grow on solid media without compromising cellular integrity. Clinically, these observations have important implications. On one hand, DTT behaves as an effective pre-analytical dispersant capable of breaking down the mucinous or biofilm-rich components commonly found in urine samples, which often impede bacterial recovery. By improving the liberation of these trapped cells, DTT helps unveil a microbial burden that culture alone may underestimate. On the other hand, FACS continues to detect viable, potentially pathogenic cells even when cultures remain negative. This aligns with the emerging understanding that a subset of “culture-negative” urinary tract infections may still harbor metabolically active bacteria that evade routine diagnostic workflows. The distribution of Δlog₁₀ differences between FACS and culture (Fig. 5) reinforces this diagnostic gap. Although DTT reduces the divergence between the two methods, the discrepancy does not disappear entirely, which may reflect the presence of viable bacterial populations that remain undetected by standard culture conditions, potentially including VBNC-like states. Taken together, these results highlight the complementary nature of combining FACS with DTT-enhanced culture. Such an integrated approach provides a more complete representation of the viable bacterial population in urine samples, improves diagnostic sensitivity, and may contribute to improved characterization of viable bacterial populations in urine samples.

**TABLE 1:** Summary of comparative analyses: summary of paired analytical comparisons between flow cytometry and plate culture, with and without DTT pre-treatment^*a*^

Comparison	N pairs	Median Δ log₁₀	Wilcoxon p	Pearson r	Spearman ρ	Interpretation
FACS vs Plate (No DTT)	69	+1.81	2.0×10⁻⁶	0.45	0.48	FACS detects viable cells not recovered by standard culture, including VBNC-like and stressed populations
FACS vs Plate (+DTT)	71	+0.50	0.0053	0.44	0.47	DTT enhances culturability and narrows the analytical gap
FACS No DTT vs FACS +DTT	72	n.s.	0.470	–^*b*^	–	DTT does not affect cytometric viability
Plate No DTT vs Plate +DTT	72	+2.74	1.6×10⁻⁵	–	–	DTT markedly increases CFU recovery

^*a*^ Values represent differences in viable counts (Δlog₁₀), statistical significance (Wilcoxon), and agreement between methods (Pearson/Spearman).

^*b*^ –, not applicable.

## DISCUSSION

This study demonstrates that low-concentration DTT (0.1%) represents a chemically compatible pre-analytical agent for urine microbiology, capable of disrupting biofilm-associated aggregates and improving bacterial culturability without altering cytometric viability. By integrating flow cytometry with culture-based enumeration, we show that DTT selectively enhances colony recovery while leaving total viable counts unchanged. This dual readout offers mechanistic insight into the discrepancy between viable but non-culturable (VBNC) bacteria and culture-based detection, providing a refined view of microbial populations in urinary infections.

DTT is widely used as a mucolytic reagent to liquefy respiratory samples and disrupt extracellular polymeric substances in biofilms. Our results confirm that 0.1% DTT does not exert detectable cytotoxic effects on urinary bacteria, as viability by FACS remained unchanged after treatment. This is clinically relevant, as higher concentrations (≥1%) have been reported to compromise membrane integrity in planktonic organisms. The optimized concentration used here therefore identifies a safe operational window for routine diagnostic use. The marked increase in CFU after DTT treatment—up to three log units in several samples—supports the concept that a substantial fraction of urinary bacteria may be entrapped within mucus, epithelial debris, or early biofilm matrices. These aggregates impair plating efficiency and contribute to reduced bacterial recovery in standard culture workflows, particularly in chronic or partially treated urinary tract infections (UTIs) (5). By dissolving these structures, DTT acts as a chemical dispersant that enhances microbial accessibility for culture and potentially for molecular assays (17).

Flow cytometry provides a culture-independent quantification of viable cells, detecting both metabolically active and quiescent forms. In untreated urine, FACS consistently exceeded culture counts, indicating the presence of viable bacterial populations not recovered by standard culture conditions, a phenomenon often associated with viable but non-culturable (VBNC) states, an established phenomenon in both environmental and clinical microbiology. VBNC cells can persist under stress conditions such as antibiotic exposure or osmotic shifts and may resume growth when conditions improve (2). After DTT exposure, the discrepancy between viable counts (FACS) and culturability (CFU) decreased but did not completely resolve. This partial convergence indicates that DTT restored growth only in a subset of non-culturable or aggregated bacteria, while a residual viable population not recovered by culture persisted, potentially including VBNC-like states. These findings underscore the complementarity of the two methods: culture captures the replicative subset, while FACS reveals the broader viable population. Combined interpretation of both may improve diagnostic accuracy in borderline or recurrent infections.

These findings have important diagnostic implications. Urinary specimens frequently contain mucinous and biofilm-derived structures capable of entrapping bacteria and limiting their detection by plate culture, particularly in patients with recurrent or partially treated UTIs. By dispersing these matrices, DTT enhances the liberation of viable pathogens that would otherwise remain undetected, thereby reducing culture underestimation in standard workflows. At the same time, flow cytometry retains its capacity to detect viable cells even when plate cultures remain negative, suggesting that some “culture-negative” symptomatic presentations may still harbor metabolically active organisms. The integrated interpretation of DTT-enhanced culture and FACS viability, therefore, provides a more complete representation of the urinary microbial burden and may improve microbiological interpretation in borderline or recurrent infections.

Beyond diagnostic performance, the clinical implications extend to vulnerable populations. These considerations may be particularly relevant in complex clinical populations, including patients with diabetes, cardiometabolic disorders, or cardiorenal disease, in whom persistent or recurrent urinary infections are frequently observed (18). This context supports the relevance of optimized pre-analytical recovery strategies in routine practice.

Concerns regarding potential contamination introduced by sample manipulation were not supported by our findings. The strengthened correlation between viable counts (FACS) and culture after DTT suggests that the increased CFU reflects recovery of the patient’s intrinsic bacterial load rather than exogenous contamination. DTT therefore appears to restore access to bacteria already present within the sample, likely those embedded in biofilm-associated aggregates.

This study benefits from paired intra-sample comparison, allowing robust assessment of DTT effects, and from the use of single-cell cytometry to quantify bacterial viability. The workflow is rapid, inexpensive, and easily compatible with routine diagnostic practice.

The study has several limitations. First, it is monocentric, which may limit the generalizability of the findings. In addition, species-level identification was not systematically performed for all culture-positive isolates, and isolates were not stratified according to Gram-positive or Gram-negative groups. Consequently, we could not evaluate whether specific uropathogens or broader bacterial groups respond differently to DTT-mediated biofilm dispersal. Flow cytometry provides quantitative information on bacterial viability but cannot determine species identity or antimicrobial susceptibility, which remain dependent on culture-based methods. Because DTT treatment occurs prior to plating and colonies grew normally on chromogenic media, no macroscopic interference with downstream identification was observed; however, formal validation of MALDI-TOF identification scores and antimicrobial susceptibility testing performance after DTT exposure will be required in future studies. Furthermore, no clinical outcome data were collected, preventing correlation of DTT-enhanced culture with symptom persistence or therapeutic response. Finally, although the discrepancy between FACS-derived viable counts and culture-based counts is consistent with the presence of VBNC-like bacterial populations, confirmatory metabolic or molecular assays (e.g., CTC staining or PMA-qPCR) were not performed and should be explored in future studies.

Future work should investigate DTT-assisted workflows in combination with MALDI-TOF, syndromic PCR panels, or metagenomics to better characterize microbial diversity within DTT-released fractions. Applications in other biofilm-rich matrices—such as respiratory secretions, synovial fluid, or wound exudates—deserve evaluation to determine the broader applicability of this approach.

In a translational perspective, this study bridges analytical and clinical microbiology. The DTT–FACS workflow preserves bacterial viability, restores culturability, and improves detection of clinically relevant pathogens. Implementing this approach in routine laboratories may improve bacterial recovery from urine samples, reduce culture underestimation, and support improved microbiological interpretation in clinical practice. By refining the identification of viable bacteria—including those contributing to low-grade inflammation—DTT-enhanced workflows may have indirect benefits for cardiometabolic and multimorbid patients, in whom urinary infections can precipitate systemic deterioration.

### Conclusion

Low-concentration DTT (0.1%) appears to be a safe and practical pre-analytical reagent that significantly enhances the sensitivity of standard urine culture by releasing bacteria entrapped within mucus or early biofilm structures. By increasing CFU recovery without altering cytometric viability, DTT narrows the gap between viable and culturable fractions and is consistent with the presence of viable bacterial populations not fully recovered by standard culture conditions, potentially including VBNC-like states in clinical urine. This workflow is inexpensive, easily implementable, and has the potential to reduce false-negative results, improve diagnostic accuracy, and support targeted therapy in urinary tract infections.

Because urinary infections may contribute to clinical deterioration in patients with diabetes and cardiometabolic/cardiorenal compromise, who represent the majority of those seen in our hospitals, improving microbial detection has implications beyond infectious disease management, supporting more comprehensive and preventive care in multimorbid populations.

Given its clinical applicability and low cost, this workflow could be evaluated for implementation in routine microbiology laboratories, including those in high-burden or resource-limited settings.

## SUPPLEMENTAL MATERIAL

Data set S1 spectrum.03910-25-s0001.docx Raw paired flow cytometry and culture data from urine samples processed with and without DTT pre-treatment.

ASM does not own the copyrights to Supplemental Material that may be linked to, or accessed through, an article. The authors have granted ASM a non-exclusive, world-wide license to publish the Supplemental Material files. Please contact the corresponding author directly for reuse.
