# Supplementary material for: Reducing culture underestimation in urine: flow cytometry–guided validation of low-dose DTT pre-treatment for biofilm dispersal
Source: Microbiol Spectr. 2026 May 5;14(6):e03910-25. doi: 10.1128/spectrum.03910-25 (PMC13228076; doi:10.1128/spectrum.03910-25)
Supplement: Data set S1 — Raw paired flow cytometry and culture data from urine samples processed with and without DTT pre-treatment. [file spectrum.03910-25-s0001.docx]

Supplementary Dataset S1. Raw paired flow cytometry and culture data from urine samples processed with and without DTT pre-treatment.

Raw dataset used for statistical analyses in this study. For each urine sample (Sample_ID), the table reports viable bacterial counts measured by flow cytometry (cells/mL) and colony-forming units determined by plate culture (CFU/mL), both before (No DTT) and after treatment with 0.1% dithiothreitol (DTT). These paired measurements were used to evaluate the effect of DTT-mediated biofilm dispersal on bacterial viability and culturability and to perform correlation and Bland–Altman agreement analyses between FACS-derived and culture-based counts.

| **Sample_ID** | **Live_ml_NoDTT** | **Live_ml_DTT** | **Plate_NoDTT** | **Plate_DTT** |
| --- | --- | --- | --- | --- |
| 2 | 333629 | 3562109 | 100000 | 1000000 |
| 3 | 457649 | 3865103 | 10000000 | 10000000 |
| 4 | 577400 | 4792510 | 10000000 | 10000000 |
| 5 | 108375 | 32986 | 100000 | 1000000 |
| 6 | 5360103 | 600321 | 10000 | 10000 |
| 7 | 4245 | 3936 | 10 | 10 |
| 8 | 1 | 3911 | 10 | 10 |
| 9 | 101075 | 586 | 10 | 10000 |
| 100 | 9438 | 7464 | 10000 | 100000 |
| 1010 | 10074 | 10470 | 10 | 10000 |
| 102 | 24105 | 2524 | 10000000 | 10000000 |
| 103 | 1067226 | 10631076 | 10000000 | 10000000 |
| 104 | 100810 | 10090 | 10 | 10 |
| 105 | 9925806 | 101010781040 | 10000000 | 10000000 |
| 106 | 3746 | 2643 | 10 | 10 |
| 107 | 51001004 | 6598210 | 10000000 | 10000000 |
| 108 | 1010210 | 669 | 10 | 10 |
| 109 | 2375 | 2807 | 10 | 10000 |
| 20 | 9962 | 1001051 | 10000000 | 10000000 |
| 210 | 56104 | 37101 | 10 | 10 |
| 22 | 5667 | 55411 | 10 | 10000 |
| 23 | 71080 | 101010746 | 10000000 | 10000000 |
| 24 | 10298 | 10464 | 10 | 10 |
| 25 | 407440 | 472587 | 10000000 | 10000000 |
| 26 | 392104 | 29095 | 10 | 10 |
| 27 | 9240 | 1010368 | 10000000 | 10000000 |
| 28 | 210035 | 105778 | 100000 | 1000000 |
| 29 | 68107 | 9928 | 100000 | 100000 |
| 30 | 6285 | 8487 | 10 | 10000 |
| 310 | 101075 | 102103 | 10 | 10 |
| 32 | 2643 | 4721 | 100000 | 1000000 |
| 33 | 68910105 | 958505 | 10 | 10 |
| 34 | 3933 | 46109 | 100000 | 1000000 |
| 35 | 1 | 1 | 10 | 10 |
| 36 | 241065356 | 23445574 | 10 | 10 |
| 37 | 10055954 | 970884 | 10000000 | 10000000 |
| 38 | 10006 | 986 | 10000000 | 10000000 |
| 39 | 764310 | 99652 | 10000000 | 10000000 |
| 40 | 6206 | 4707 | 10 | 100000 |
| 410 | 269101026 | 21089065 | 1000000 | 10000000 |
| 42 | 4409 | 400 | 10 | 10 |
| 43 | 5659677 | 341010529 | 10 | 10 |
| 44 | 380 | 387 | 100000 | 1000000 |
| 45 | 810490 | 50332 | 10 | 10000 |
| 46 | 7497 | 5984 | 10 | 10 |
| 47 | 1 | 1000 | 10 | 10 |
| 48 | 23684 | 29340 | 10000000 | 10000000 |
| 49 | 498745 | 4100357 | 10000000 | 10000000 |
| 50 | 63811 | 36100 | 100 | 1000000 |
| 510 | 4487 | 46104 | 100 | 100 |
| 52 | 598 | 399 | 100 | 10000 |
| 53 | 2286 | 2056 | 100 | 100 |
| 54 | 8331 | 7955 | 100 | 100 |
| 56 | 101058 | 10843 | 100 | 100000 |
| 57 | 299 | 399 | 100 | 100 |
| 58 | 684550 | 4510541 | 100000 | 10000000 |
| 59 | 105810 | 394 | 100 | 100000 |
| 60 | 678475 | 101034667 | 1000000 | 10000000 |
| 610 | 48489 | 45449 | 100 | 100000 |
| 62 | 7403 | 7490 | 100 | 100 |
| 63 | 28686 | 256911 | 100 | 100 |
| 64 | 2053 | 210311 | 100 | 100 |
| 65 | 635101 | 758010 | 1000000 | 10000000 |
| 66 | 27769 | 1039811 | 100 | 100 |
| 67 | 24937 | 4210 | 10000000 | 10000000 |
| 68 | 10321010 | 1010469 | 100 | 100 |
| 69 | 583 | 398 | 100 | 100 |
| 70 | 2594 | 10396 | 100 | 100 |
| 710 | 6084 | 6846 | 10000 | 100000 |
| 72 | 300881 | 294508 | 100000 | 100000 |
| 73 | 4007864 | 401006100 | 10000000 | 10000000 |
| 74 | 7101085 | 78384 | 100 | 100 |
